# Leukocyte adhesion deficiency syndrome: report on the first case in Chile and South America

**DOI:** 10.1590/S1516-31802012000400011

**Published:** 2012-09-04

**Authors:** Rodrigo Vásquez-De Kartzow, Cristian Jesam, Valentina Nehgme, Francisco Várgas, Carolina Sepúlveda

**Affiliations:** I MD. Specialist in Pediatrics and Infectious Diseases, Department of Pediatrics, Campus Centro, Facultad de Medicina de la Universidad de Chile, Santiago, Chile.; II MD. General Physician, Universidad de Los Andes (ULA), Santiago, Chile.; III MD. Pediatrician, Hospital Parroquial de San Bernardo (HPSB), Santiago, Chile.; IV MD. Pediatrician, Universidad de Los Andes (ULA), Santiago, Chile.

**Keywords:** Child, Immunologic deficiency syndromes, Case report [publication type], Cell adhesion molecules, Leukocyte-adhesion deficiency syndrome, Niño, Síndromes de inmunodeficiencia, Informes de casos, Moléculas de adhesión celular, Síndrome de deficiencia de adhesión del leucocito

## Abstract

**CONTEXT:**

Adhesion molecule deficiency type 1 is a rare disease that should be suspected in any patient whose umbilical cord presents delay in falling off, and who presents recurrent severe infections. Early diagnostic suspicion and early treatment improve the prognosis.

**CASE REPORT:**

The case of a four-month-old boy with recurrent hospitalizations because of severe bronchopneumonia and several episodes of acute otitis media with non-purulent drainage of mucus and positive bacterial cultures is presented. His medical history included neonatal sepsis and delayed umbilical cord detachment. Laboratory studies showed marked leukocytosis with predominance of neutrophils and decreased CD11b and CD18. These were all compatible with a diagnosis of leukocyte adhesion deficiency type I [LAD type 1].

## RESUMO

**CONTEXTO:**

El deficit de moleculas de adhesión tipo 1 es una enfermedad rara que debe ser sospechada en todo paciente que presente un retardo en la caída del cordón unmbilical, además de infecciones graves a repetición. Un sospecha diagnóstica precoz y un tratamiento oportuno mejorarán el pronóstico.

**INFORMES DE CASO:**

Se presenta el caso de un niño de cuatro meses de edad, con hospitalizaciones recurrentes a causa de bronconeumonía severa y varios episodios de otitis media aguda con drenaje mucoso, no purulento, y cultivos bacterianos positivos. Su historial médico incluye la sepsis neonatal y el desprendimiento tardío del cordón umbilical. Los estudios de laboratorio mostraron leucocitosis marcada con predominio neutrofílico y disminución de CD11b y CD18, todos compatibles con el diagnóstico del tipo de deficiencia de adhesión leucocitaria 1 [tipo LAD 1].

## INTRODUCTION

Leukocyte adhesion deficiency (LAD) type 1 is a rare disease with only 200 cases reported in the medical literature.^1^ It was first described in 1970,^2^ but no cases have been described in Latin America. It is clinically suspected in infants with a history of delayed umbilical cord detachment (after day 15)^3^ and recurrent bacterial/fungal cutaneous and mucosal infections characterized by absence of pus formation and eventual septic shock and death.^2^^,^^3^^,^^4^

We report the case of an infant with LAD type 1 who was diagnosed at the Pediatric Service of Hospital Parroquial de San Bernardo, in Santiago, Chile, and present a review of the medical literature.

## CASE REPORT

A four-month-old male infant born in October 2001, who was the only child of consanguineous parents, was admitted to the intensive care unit of our hospital with diagnoses of septic shock, bilateral suppurative otitis media, thrush and diaper area candidiasis.

Three days prior to admission, he had developed fever that partially responded to acetaminophen. The physical examination revealed tachycardia, weak peripheral perfusion, significant weight loss, hepatosplenomegaly, abundant mucous discharge from both tympanic ducts, erythematosquamous cutaneous lesions on the thoracic wall, thrush and diaper area dermatitis.

His blood pressure recovered soon after admission, through rapid infusion of intravenous fluids and transfusion of red blood cells. Intravenous cefotaxime and cloxacillin were started.

The laboratory tests at the time of admission revealed leukocytosis with neutrophil predominance (> 40 x 10^3^/mm^3^) that persisted during this hospitalization in spite of the antibiotic treatment. The chest radiography showed mild, bilateral, symmetrical interstitial infiltrates. Urine and cerebrospinal fluid (CSF) cytochemistry were normal. Blood, urine and CSF cultures were sterile. Otic discharge culturing showed the presence of *Haemophilus influenzae* type B and coagulase-negative *Staphylococcus*. The antibiotic treatment was maintained for 14 days, with a good clinical response.

The patient’s past medical history consisted of uncomplicated fekal development with full-term eutocic delivery and weight of 3,400 grams. He had respiratory depression and sepsis of unknown origin at birth and was treated empirically with broad-spectrum antibiotics. His umbilical cord detached late, at 21 days of age, and since day 23, he had presented recurrent episodes of bilateral non-purulent otitis media that were resistant to regular antibiotic treatment.

He had been readmitted at the age of two months because of bronchopneumonia and mucoid suppurative otitis media. During that hospital stay, he was treated for cellulitis that originated at venipuncture sites and affected his entire arm. He was treated with intravenous penicillin and cloxacillin with a good outcome.

On the basis of the recurrent infections and physical findings, a diagnosis of primary immunodeficiency was suggested. An ELISA test for human immunodeficiency virus and tests for complement components and quantitative immunoglobulin isotypes A, G, M and E were done. In addition, determinations of CD4, CD8 and the CD4/CD8 ratio were all normal. Flow cytometry analysis for CD18 and CD11 were requested, and also bone images. Finally, a bone marrow biopsy was done to rule out histiocytosis.

**Figure 1.**
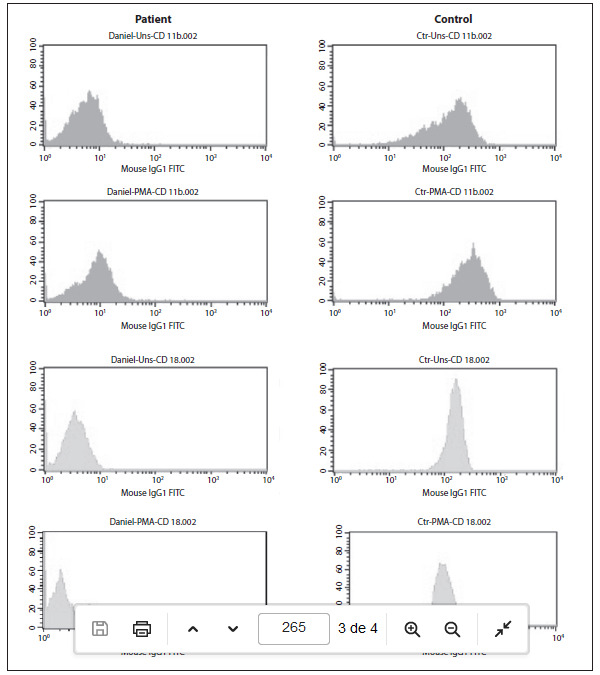
Flow cytometry analysis. Detection of CD18 expression in peripheral blood neutrophils and lymphocytes, in the LAD-1 patient and a healthy control. The patient had small populations of CD11b/CD18+ cells (9% and 3% respectively). PMA= phorbol myristate acetate.

The diagnosis of leukocyte adhesion deficiency (LAD) type 1 was suspected because of the persistent leukocytosis and the history of a delay in umbilical cord detachment. Because of this suspicion, the patient was started on prophylactic oral therapy with amoxicillin/clavulanate and nystatin, and was discharged. A flow cytometry analysis, kindly provided by Louisiana State University Health Sciences Center/Children’s Hospital Immunology Laboratory, detected that the CD11 and CD18 levels were low, thus confirming the diagnosis of LAD type 1 (Figure 1). Bone marrow or stem cell transplant was not available, and the patient died at the age of four years at another city hospital because of severe sepsis.

## DISCUSSION

Congenital immunodeficiency is generally suspected during early infancy. Such conditions may be classified into: 1) primary defects of antibody production, which represent approximately 50% of such cases (X-chromosome-linked agammaglobulinemia, selective deficit of immunoglobulins IgA and IgG, hyper-IgM, common variable hypergammaglobulinemia, specific antibody deficit and transient hypogammaglobulinemia); 2) combined immunodeficiency, accounting for 20% (combined severe immunodeficiency and immunodeficiency relating to other defects like Wiskott-Aldrich and ataxia telangiectasia); and 3) congenital diseases of phagocytes and other natural immunity cells, accounting for 18%. Of these, primary defects of T lymphocytes are responsible for 10%. Adhesion molecule deficiencies are extremely rare.^5^

Pathogenically, LAD is characterized by loss of the leukocytes’ ability to adhere to the endothelium during the inflammatory cascade, thus preventing their migration to the infected tissues. In a normal person, leukocytes first adhere to and roll along the wall of blood vessels through using selectins and glycoconjugate ligands. They then adhere firmly and transmigrate using integrins and endothelial ligands. Total or partial absence of these molecules leads to LAD.^6^

Adhesion molecule deficiencies have been classified into two types: LAD-1 and LAD-2, depending on the molecule that is defective, i.e. integrins in LAD-1 and selectins in LAD-2. Both forms of LAD have an autosomal recessive transmission pattern.^4^

Molecularly, integrins are heterodimeric protein receptors composed of a and b polypeptide subunits. These subunits combine in humans to produce at least 24 different heterodimers through non-covalent associations between a beta subunit and an alpha subunit.

LAD-1 consists of failure to express the aMb2 and aLb2 integrins, which serve as the receptor for C3b in myeloid and lymphoid cells. These integrins are encoded by the CD18 gene, which is mapped to the long arm of chromosome 21. LAD-1 has a variable phenotype and may be classified as severe, moderate or variable, depending on the leukocytes’ level of CD18 expression. In the severe form, there is almost total absence of CD18 in leukocytes (< 1%), and such conditions usually end in early death. In the moderate form, the CD18 expression level is 2% to 5%, with moderate persistent leukocytosis, which results in less frequent infections and a better survival rate. Finally, in the variable form, normal levels of CD18 are expressed, but they are non-functional.

LAD-2 is less common and was described for the first time in 1992 in Palestinian children born from unrelated parents.^7^ Its symptoms are similar to LAD-1, but children with LAD-2 are usually mentally retarded and present failure to thrive, delayed growth, dysmorphic phenotype characteristics and a Bombay blood phenotype.^4^^,^^7^ These infants are unable to synthesize fucose from GDP mannose and therefore do not form the sialyl-Lewis x (sLex) ligand for the selectin molecules of the surface of neutrophils.^8^^,^^9^ Cell analysis on these infants shows a change in the GDP-fucose transporter from the cytoplasm to the Golgi apparatus.^4^ This is the reason why cell adherence mediated by E and P selectins is seriously compromised in these patients.

Patients with LAD-1 typically present recurrent bacterial and fungal infections of the skin and mucosa, delayed detachment of the umbilical cord, rapidly progressive periodontitis, osteomyelitis and, less commonly, hepatosplenomegaly.^2^^,^^10^^,^^11^^,^^12^ The diagnosis is suggested by the described findings, associated with persistent leukocytosis with neutrophil predominance. Flow cytometry is diagnostic and will show a notable decrease or absence of CD18.^2^^,^^4^

Bone marrow or stem cell transplant has been shown to improve the survival of LAD-1 patients.^13^ The prognosis for patients with the severe form of LAD-1, like the patient presented here, is poor. Most of them will die before reaching 10 years of age if a transplant cannot be performed. Patients with CD18 levels between 1 and 10% may live to the age of 40 years or more.^7^ Use of oral fucose could be a novel alternative for patients with LAD-2.^8^ Gene therapy studies are evaluating promising treatments for these disorders.^2^

We reviewed the literature in Medline and PubMed, using the keywords CD11/CD18 - adhesion molecule deficiencies. We found that few cases of the deficiency of CD11/CD18 adhesion molecules presented by our patient had been reported. For this reason, it seems important to contribute through the present paper, towards disseminating greater understanding of this condition (Table 1).

**Table 1.** Total numbers of papers indexed in the Lilacs (Literatura Latino-Americana e do Caribe em Ciências da Saúde) and PubMed databases between 1980 and 2011. Search performed on September 6, 2011

Database	Search strategy (descriptors)	Results
Lilacs	Adhesion celular (DeCs)	126 articles
	Síndrome de Deficiencia de Adhesión del Leucocito (DeCs)	2 articles
	Síndrome de Deficiencia de Adhesión del Leucocito E niños (DeCs)	5 articles
PubMed	Leukocyte-Adhesion Deficiency Syndrome (MeSH)	201 articles
	Leukocyte-Adhesion Deficiency Syndrome AND Children (MeSH)	36 articles
	Cell Adhesion molecules (MeSH)	5 articles

## CONCLUSIONS

The importance of suspecting and diagnosing this disease lies in the possibility of offering early treatment for possible infections, and prophylactic measures to improve the prognosis.
